# EDISA: extracting biclusters from multiple time-series of gene expression profiles

**DOI:** 10.1186/1471-2105-8-334

**Published:** 2007-09-12

**Authors:** Jochen Supper, Martin Strauch, Dierk Wanke, Klaus Harter, Andreas Zell

**Affiliations:** 1 Center for Bioinformatics Tübingen (ZBIT), University of Tübingen, Sand 1, 72076 Tübingen, Germany; 2 Center for Plant Molecular Biology (ZMBP), University of Tübingen, Auf der Morgenstelle 1, 72076 Tübingen, Germany

## Abstract

**Background:**

Cells dynamically adapt their gene expression patterns in response to various stimuli. This response is orchestrated into a number of gene expression modules consisting of co-regulated genes. A growing pool of publicly available microarray datasets allows the identification of modules by monitoring expression changes over time. These time-series datasets can be searched for gene expression modules by one of the many clustering methods published to date. For an integrative analysis, several time-series datasets can be joined into a three-dimensional *gene-condition-time *dataset, to which standard clustering or biclustering methods are, however, not applicable. We thus devise a probabilistic clustering algorithm for *gene-condition-time *datasets.

**Results:**

In this work, we present the EDISA (Extended Dimension Iterative Signature Algorithm), a novel probabilistic clustering approach for 3D *gene-condition-time *datasets. Based on mathematical definitions of gene expression modules, the EDISA samples initial modules from the dataset which are then refined by removing genes and conditions until they comply with the module definition. A subsequent extension step ensures gene and condition maximality. We applied the algorithm to a synthetic dataset and were able to successfully recover the implanted modules over a range of background noise intensities. Analysis of microarray datasets has lead us to define three biologically relevant module types: 1) We found modules with independent response profiles to be the most prevalent ones. These modules comprise genes which are co-regulated under several conditions, yet with a different response pattern under each condition. 2) Coherent modules with similar responses under all conditions occurred frequently, too, and were often contained within these modules. 3) A third module type, which covers a response specific to a single condition was also detected, but rarely. All of these modules are essentially different types of biclusters.

**Conclusion:**

We successfully applied the EDISA to different 3D datasets. While previous studies were mostly aimed at detecting coherent modules only, our results show that coherent responses are often part of a more general module type with independent response profiles under different conditions. Our approach thus allows for a more comprehensive view of the gene expression response. After subsequent analysis of the resulting modules, the EDISA helped to shed light on the global organization of transcriptional control. An implementation of the algorithm is available at http://www-ra.informatik.uni-tuebingen.de/software/IAGEN/.

## Background

Cellular signaling events affect the regulation of transcription factor (TF) activation [1,2]. TFs in turn regulate the expression of specific target genes. Microarrays can provide dynamic information on the phenomenological responses induced by this underlying regulatory network. Such datasets are either analyzed by approaches explicitly modeling regulatory interactions [3-5] or are clustered into co-expressed groups of genes, which potentially capture genes under the same regulatory control [6-8]. Both approaches have been extended to integrate homogeneous [9] or heterogeneous [5,8] information potentially leading to more expressive models. In this work we concentrate on the clustering paradigm in order to devise an integrative approach for application to homogeneous datasets.

The majority of DNA microarray assays monitor the expression of genes over several time-points or conditions, providing a two-dimensional dataset. Such datasets are often processed by full-space clustering approaches, such as *k*-means clustering [10], hierarchical clustering [11], and spectral clustering [12]. In 2000, the biclustering approach was introduced by Cheng *et al*. [13]. Further publications followed [8,14-18]. These biclustering methods aim at finding subsets of genes and conditions by clustering them simultaneously.

For an integrative analysis of 3D *gene-condition-time *datasets with standard clustering or biclustering approaches, these datasets have often been projected onto a single *gene-condition *matrix, with each time-point labeled as a separate condition [14,19,20]. However, these approaches ignore the time-dependent structure of the dataset, directly comparing expression values from different experiments. Hence, the variation within the dataset and the number of potential modules increase. To analyze multiple time-series datasets without disrupting their structure, Zhao and Zaki [21] recently proposed the TRICLUSTER approach. This approach extends the concept of biclustering by an additional dimension. Daxin *et al*. [9] introduced two algorithms extending the idea of a full-space clustering approach. These methods mine for genes that have coherent patterns across both the condition and time dimension, hence *coherent *modules. Such modules impose a strong constraint on the dataset, which has to be equidistant in its time-steps, and onthe biological response trajectory, which has to follow the same shape under every condition.

An important prerequisite for such approaches is the availability and composition of three-dimensional *gene-condition-time *datasets. Such datasets could be composed as an accumulation of different experiments from microarray databases [22-24], leading to a heterogeneous dataset. Here, however, we concentrate on homogeneous datasets generated within one study. For our analysis, we used a multiple sclerosis dataset from *Homo sapiens *and two datasets from *Arabidopsis thaliana*. In 2003 a 3D dataset from multiple sclerosis patients [25] has been published. The condition dimension consisted of 13 multiple sclerosis patients, monitored over 7 time-points after IFN-*β *injection. The *Arabidopsis thaliana *datasets were composed of different abiotic stress stimulus experiments conducted in the root and shoot tissue [26]. This dataset has been previously analyzed by methods which cluster each condition separately [17,27] and by other approaches [28,29] employing different standard methodologies to provide a comprehensive biological interpretation of the datasets.

In order to mine 3D *gene-condition-time *datasets with different module definitions we established the EDISA (Extended Dimension Iterative Signature Algorithm), which is based on the Iterative Signature Algorithm (ISA) proposed by Bergman *et al*. [30] in 2003. We chose to extend the ISA algorithm because it was successfully applied to *Saccharomyces cerevisiae *microarray data [31], ranked among the best biclustering algorithms in a comparative study [20], and was flexible enough to be extended by a further dimension and novel module definitions. However, a acknowledged problem of the ISA lies in its predilection for strong signals, which are found hundreds of times before weaker signals are, if at all, detected. In cases where genes with a strong signal have been selected into the initial sample, they dominate the average, driving the module towards their signal. Lazzeroni and Owen [32] address a similar problem by subtracting signals which are contained in the already detected modules. Kloster *et al*. [33] extend the ISA, stipulating that the condition vector of each new module be orthogonal to the condition vectors of the previously identified modules.

We borrowed the idea of iteratively refining the genes and conditions contained in a module from the ISA. However, the module definitions as well as the pre- and postprocessing steps were redesigned and further module definitions were added. The preprocessing was redesigned to compensate for the predilection of the ISA approach for strong signals, whereas the postprocessing was designed to generate a comprehensive set of distinct modules.

## Results and discussion

### Approach

The method proposed in this work is the Extended Dimension Iterative Signature Algorithm (EDISA). It is an extension of the ISA approach [30]. While the ISA operates on two-dimensional datasets, the EDISA is capable of mining gene modules in the three-dimensional datasets used throughout this work.

Commonly, two-dimensional gene expression datasets comprise a gene and a condition dimension. Thus, to extract modules, biclustering algorithms mine for subsets of genes and conditions within a permutable matrix of expression values. In the case of a three-dimensional dataset, the *gene-condition *matrix contains time-series of gene expression values, rather than scalar values.

**Figure 1:**
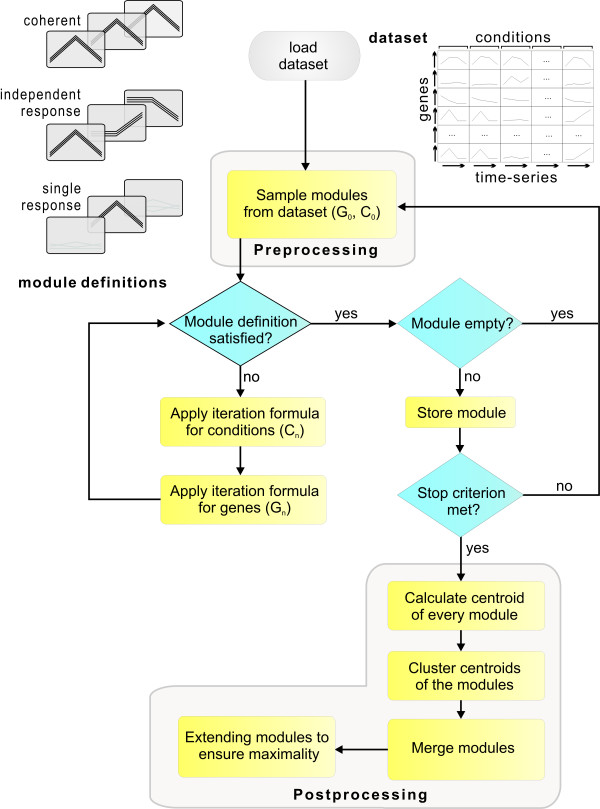
**Schematic flowchart depicting the EDISA**. Before applying EDISA, the module definition and the dataset have to be specified. Here, we provide three predefined module types. Given this information random samples are drawn from the dataset (preprocessing). EDISA iteratively refines these samples and stores them if they match the module definition. After a specified number of runs EDISA computes the final modules (postprocessing).

For each module type, a mathematical definition is provided in the methods section, specifying the set of all modules of a particular type contained in a dataset. To mine for all modules, one could, in principle, enumerate all the subsets of genes and conditions. This, however, is intractable as the number of possible subsets grows exponentially with the genes and conditions. Nonetheless, we want to mine the datasets for all modules contained therein in reasonable time. To accomplish this, several steps are taken. We introduce a simple probabilistic preprocessing method to sample subsets of genes potentially containing a module. Typically, the number of conditions is small compared to the number of genes. Thus, we can include all the conditions into the initial module and only sample the genes. Starting from initial samples, the EDISA algorithm refines sets of genes and conditions at each iteration step. This is achieved by removing genes and conditions not sufficiently aligned with the average expression pattern of the module. The iteration formulas are repeated until convergence is reached. Convergence is reached if the module definition is satisfied for all genes and conditions. If a module is found, it is stored and the procedure is repeated, leading to a redundant set of modules. The postprocessing step merges these modules into a non-redundant set. This reduction is accomplished by removing modules that are a subset of a larger module. Finally, for every remaining module, two extension steps are applied, ensuring gene and condition maximality. A schematic overview of the EDISA algorithm is given in Figure 1.

### Gene modules

Our definitions of gene modules are designed to fulfill several conditions, that enable us to capture the modular structure of transcriptional control [7,34]. The modules are non-exhaustive, since some genes might be unaffected under the conducted experiment. They are non-exclusive, since a gene might be regulated by different mechanisms under different conditions. To account for this, it is possible to assign a gene to multiple modules. Apart from the general concept of a gene module, each module definition provided here is derived from a particular biological intuition. *Single response *modules associate genes with one condition, uncovering very specific response mechanisms that may help biologists to find marker genes for certain stresses. *Coherent *modules, on the other hand, reveal co-expression under multiple conditions and display a more general response. The genes involved in both the *single response *and *coherent *modules are potentially controlled by the same transcription factors. *Independent response *modules allow for a more complex type of modular co-expression, i.e. they hint at the existence of stress responses specific for every condition alongside with a common transcriptional control. The introduction of *independent response *modules extends previous approaches by a novel module type [9,21].

### Parameter settings

In order to obtain correlated biclusters on biological datasets, it is essential to find the threshold which is able to distinguish different signals while separating them from noise. EDISA has two main parameters, *τ*_*G *_and *τ*_*C*_. The *τ*_*G *_parameter specifies how well each gene has to be aligned with the average trajectory of the module. Respectively, parameter *τ*_*C *_specifies how well each condition has to be aligned with the average trajectory of the module. Conceptually, *τ*_*G *_can be related to the intra-condition variance of different genes under the same regulatory control, and *τ*_*C *_can be related to the inter-condition variance of a gene.

A common strategy for adjusting these parameters, which is already known from the ISA [30], requires several passes over the data at different resolutions. Low values of *τ *will create modules containing highly correlated gene expression profiles. Hence, increasing the value of *τ *will result in modules containing an increasing number of genes, that display a reduced correlation. Here, we adjust parameters *τ*_*G *_and *τ*_*C *_during the process of extracting the modules. The adjustment is based on a clustering step which separates the signals from noise (for details, see Automatically refining the parameters). In a postprocessing step the obtained modules are matched against their module definition, for which fixed *τ*_*G *_and *τ*_*C *_parameters are applied.

Thus, the user can specify three parameters *τ*_*G*_, *τ*_*C *_and the number of iterations performed, i.e. the number of samples drawn from the dataset.

### Testing

In order to evaluate its performance, the EDISA was applied to a randomly generated synthetic dataset. Therefore, the number of samples was set to 1,000, *τ*_*G *_to 0.1 and *τ*_*C *_to 0.2, for the noise levels *σ *∈ [0, 0.5]. For the noise level *σ *= 0.7 the parameter *τ*_*G *_was set to 0.15 and for the noise level *σ *= 0.9 the parameter *τ*_*G *_was set to 0.2. The synthetic dataset contains three overlapping modules and one exclusive module.

**Figure 2:**
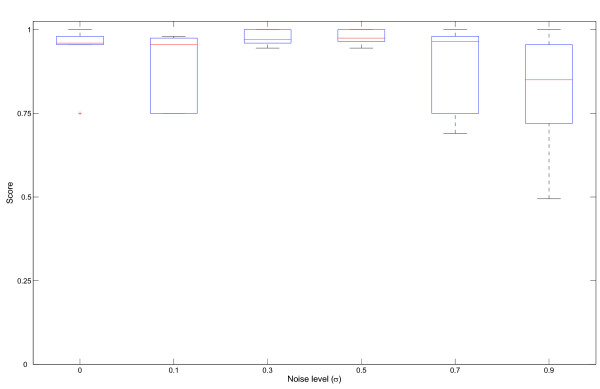
**Performance of EDISA on an synthetic dataset**. EDISA was applied to a synthetic dataset with implanted modules and different levels of noise. The overlap of the implanted modules and the modules mined by EDISA were scored (equation 15). Six runs with 400 iterations were performed, with *τ*_*G *_= 0.1 and *τ*_*C *_= 0.2 for *σ *∈ [0,0.5], *τ*_*G *_= 0.15 for *σ *= 0.7 and *τ*_*G *_= 0.2 for *σ *= 0.9.

Apparently, EDISA is robust against noise applied to the modules and the variance of the results from separate runs is fairly small (Figure 2), given the probabilistic nature of the method. A direct comparison of the EDISA to the methods of Zhao and Zaki [21] and Daxin *et al*. [9] is not provided here, since such a comparison could only be carried out for *coherent *modules.

### Application to biological datasets

EDISA has been applied to the biological datasets to mine for *coherent *and *independent response *modules. We did not explicitly mine *single response *modules, since they are conceptually contained in *independent response *modules [see Additional file 2]. On the biological datasets the EDISA was applied with 10,000 iterations and the threshold settings *τ*_*G *_= 0.1 and *τ*_*C *_= 0.2. An overview of the obtained modules is given in Table 1.

**Table 1:** Modules found in the datasets

	**modules**		
datasets	*coherent*	*independent response*	*single response*
*Homo sapiens *multiple sclerosis	8	15	0
*Arabidopsis thaliana *root	5	34	8
*Arabidopsis thaliana *shoot	13	47	5

Number of modules and module types found in each dataset after searching for *coherent *modules and *independent response *modules separately. The *single response *modules are a subset of the *independent response *modules. In each case 10,000 EDISA iterations were performed with *τ*_*G *_= 0.1 and *τ*_*C *_= 0.2.

To gain insight into the ability of the EDISA to produce biological relevant models, we related the obtained modules to their biological process, by mapping the respective genes to the Gene Ontology (GO) [35]. This mapping was performed with DAVID [36]. DAVID calculates *p*-values by employing a modified Fisher Exact test (EASE) [37], which is based on the hypergeometric distribution. Additionally, for the *Arabidopsis thaliana *dataset we performed an enrichment analysis for *cis*-regulatory motifs.

### Evaluation on the Homo sapiens multiple sclerosis dataset

**Figure 3:**
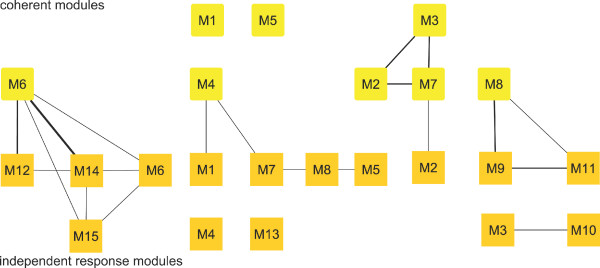
**Overview of the Homo sapiens multiple sclerosis modules**. Depicts the *independent response *and *coherent *modules. The edges indicate the amount of overlap between the modules (equation 14), if the respective value is lower than 0.15 no line is drawn. Table 1 provides an overview of all different module types.

**Figure 4:**
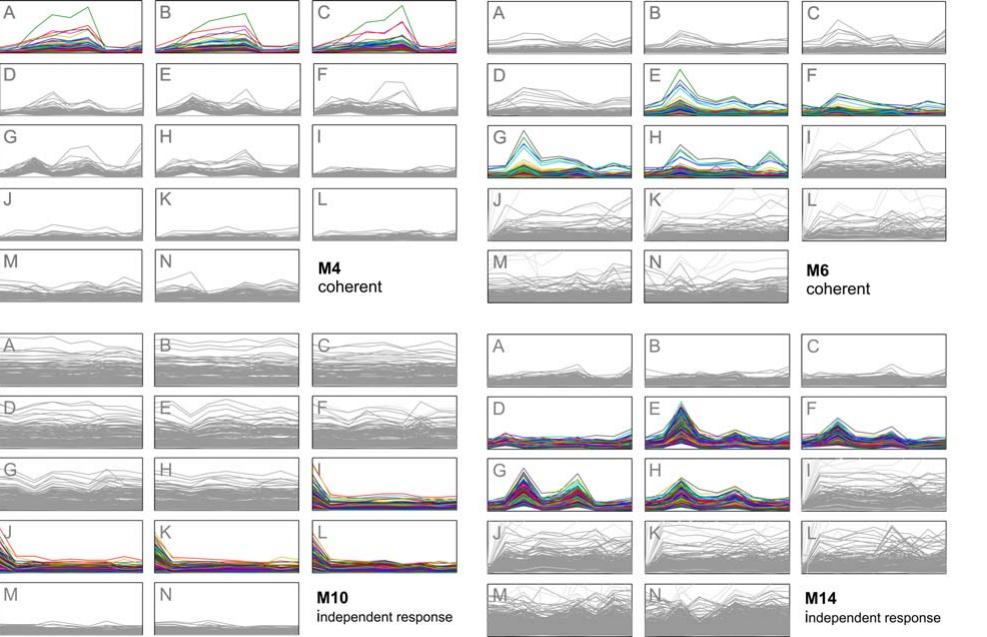
**Modules in the Homo sapiens multiple sclerosis dataset**. The modules of the *Homo sapiens *dataset show distinct responses for the different patients. Module M4 depicts a response of patients A-C. Modules M6 and M14 cover a similar response trajectory, while M4 was detected with the *coherent *definition and M14 with the *independent response *definition. Module M10 is associated with the patients I-J and depicts a group of genes which are down-regulated after IFN-*β *treatment. Conditions not contained in a module are depicted in gray.

The *Homo sapiens *multiple sclerosis dataset was obtained from multiple sclerosis patients after injection of 30 *μg *of IFN-*β*, with GeneFilters GF211 DNA arrays. After applying the EDISA to this dataset, we received 15 *independent response *modules (M_*IR*_)^, ^8 *coherent *modules (M_*Co*_) but no *single response *modules (M_*SR*_) (Figure 3). These modules capture 2,420 different genes responding to the IFN-*β *treatment [38]. Several modules cover responses in which the genes of patients I-L are down-regulated (e.g. M10_*IR*_, Figure 4) or up-regulated (e.g. M13_*Co*_), respectively. Another group of modules exists which captures peaked responses affecting the genes of patients E-H (M6_*Co *_and M14_*IR*_, Figure 4). Analysis with the functional Gene Ontology annotation often showed an enrichment for the regulation of cellular processes such as the regulation of the nucleic acid and protein metabolism. As IFN-*β *is known to inhibit proliferative activities [39], the functional enrichment of cell growth and/or maintenance is plausible. The extracted modules often reveal striking differences between the patients of this study. For instance the patients A-C are associated with the modules M3_*Co*_, M4_*Co*_, and M7_*Co*_. Whereas, the patients I-L are associated with the modules M8_*Co *_and M9_*IR*_-M11_*IR*_.

This grouping allows a phenomenological classification of patients with respect to their stress responses. For instance, only patients E-H are associated with module M6_*Co *_with a functional enrichment of "cell cycle" (*p*-value: 1.0 *× *10^-5^) and "organic acid metabolism" (*p*-value: 7.3 *× *10^-4^). Whereas, only patients I-L are associated with the module M10_*IR*_, with a functional enrichment of "response to stress" (*p*-value: 7.0 *× *10^-4^). These response characteristics can be related to independent differences in disease history or progression.

*Single response *modules were not observed as could be expected since the external stimulus is identical for all patients. Surprisingly, this did not result in a high number of *coherent *modules. Instead, we observed a rather high number of *independent response *modules.

### Evaluation on the Arabidopsis thaliana abiotic stress dataset

To analyze the response to several abiotic stresses, a comprehensive *Arabidopsis thaliana *transcript expression study was performed for the tissues root and shoot [26]. These measurements were performed in parallel on Affymetrix ATH1 microarrays. Some time-points in this dataset were not consistently measured under all conditions, and have therefore, been removed when mining for *coherent *modules.

**Figure 5:**
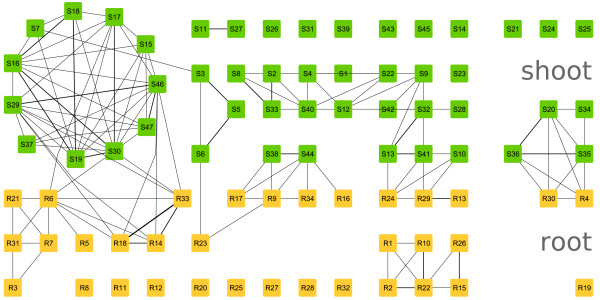
**Overview of the Arabidopsis thaliana modules**. Depicts the *independent response *modules. The edges indicate the amount of overlap between the modules (equation 14). If the respective value is lower than 0.15 no line is drawn. Table 1 provides an overview of all different module types.

Employing the EDISA, we extracted 47 *independent response *modules from the shoot (S1_*IR*_-S47_*IR*_, Figure 5) and 34 *independent response *modules from the root dataset (R1_*IR*_-R34_*IR*_, Figure 5). From 4,491 genes, which meet the fold-change criteria, 2,617 were included into at least one module. The modules can be grouped into four components, explaining most of the variation in gene expression. These four components are: The circadian rhythm, the heat shock response, a general stress response and specific stress responses.

#### Circadian rhythm

**Figure 6:**
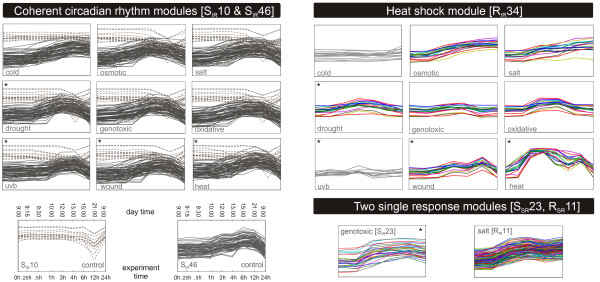
**Module types in Arabidopsis thaliana**. The circadian rhythm modules S10_*IR *_(dotted) and the S46_*IR *_(solid) are examples for modules with a coherent trajectory over all conditions, except cold. For both modules the control measurements are depicted, along with the time-points of the experiments and the respective time of the day. The heat shock module (S23_*IR*_) is an *independent response *module, with a strong signal under heat. Various profiles can be observed along with a clear co-expression. The two *single response *modules S23_*IR *_and R11_*IR *_are depicted only with the condition under which the response pattern can be observed. Conditions marked with a star (*) comprise transient stresses, all other stresses were applied permanently. Conditions not contained in a module are depicted in gray.

The circadian rhythm in the shoot tissue is an excellent example of a coherent response, displayed by the modules S10_*IR *_and S46_*IR *_(Figure 6). The genes of module S46_*IR *_are up-regulated at daytime, and the genes of S10_*IR *_are down-regulated at daytime. Unlike the genes of other modules, the circadian clock genes do not respond to the applied stress, since the pattern of S46_*IR *_can also be observed in the control measurement of the shoot tissue. Under cold stress, however, this oscillation is disrupted. Gould *et al*. [40] describe how the *Arabidopsis thaliana *circadian clock usually compensates for temperature differences. They cover a temperature range from 12 to 27°, whereas a cold stress of 4° was applied here. Ramos *et al*. [41] discovered that two chestnut proteins (CsTOC1 and CsLHY), which are homologous to two proteins of the circadian rhythm in *Arabidopsis thaliana*, fail to oscillate during winter dormancy. This supports the finding of a clock disruption under cold stress.

For the S46_*IR *_module we could identify two *cis*-regulatory elements that are highly enriched in the upstream regulatory sequences, a GCCAC motif (*p*-value: 2.5 *× *10^-1^) and the consensus for the well known G-box, CACGTG (*p*-value: 4.3 *× *10^-5^). Although the first motif is of low significance, it is noteworthy that both motifs have already been discovered in two publications on phytochrome mediated light signaling cascades [42]. The G-box is bound by specific G-box-binding transcription factors of the bZIP-transcription factor family and has already been shown to constitute an essential regulatory element in several promoters of light regulated genes.

#### Heat shock response

A clear example of an *independent response *module can be observed in the module R34_*IR *_consisting of 17 genes (Figure 6). Of these, 9 map to "response to heat" (*p*-value: 2.0 *× *10^-17^), 13 to "response to abiotic stimulus" (*p*-value: 8.5 *× *10^-15^) and 7 map to "protein folding" (*p*-value: 7.0 *× *10^-9^). Apparently, this module is mainly composed of heat shock proteins which have already been described to be co-regulated by the heat shock transcription factors hsf1 and hsf3 [43]. Both factors bind to the spaced dyad palindrome GAA(N)TTC. Indeed, this motif occurs frequently in the upstream sequences of the genes in this module (*p*-value: 4.9 *× *10^-06^).

#### Cold, osmotic, and salt stress response

**Figure 7:**
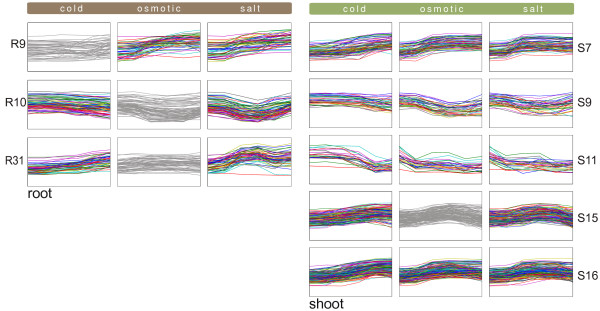
**General stress responses in Arabidopsis thaliana**. EDISA identified numerous modules comprising two or three conditions out of the set cold, osmotic, salt. As these signals belong to the most common in the whole dataset, an overview of the responses for these three conditions is given here, whereas modules with strong responses under the other conditions are omitted. The left column depicts modules found in the root tissue and the right column those found in the shoot tissue. Generally, the responses to osmotic and salt stress are very similar in shape. All conditions shown here comprise permanent stresses. Conditions not contained in a module are depicted in gray.

The most prevalent signals appear under the cold, osmotic and salt conditions (Figure 7). Kreps *et al*. [44] found about one third of the Arabidopsis genome to be sensitive to these three conditions. EDISA detects several modules taking part in the response to these stresses. Several distinct shapes can be observed, which are similar for salt and osmotic stress. This suggests that genes are co-regulated under osmotic and salt stress, or, more likely, that the plant does not distinguish between salt and osmotic stress most of the time. Overall, these modules seem to be the result of an underlying general stress response mechanism, which is activated by different stresses. Module S7_*IR *_is significant for "response to water deprivation" (*p*-value: 2.9 *× *10^-8^) and is pronounced under "response to cold" (*p*-value: 1.6 *× *10^-16^). Module S16_*IR *_is assigned to "response to cold" (*p*-value: 1.6 *× *10^-16^), "response to salt stress" (*p*-value: 1.0 *× *10^-5^) and " response to osmotic stress" (*p*-value: 3.3 *× *10^-5^) confirming the cold, osmotic and salt component of this module. In the promoters of the genes contained within this module we found the drought and cold responsive element ACCGAC enriched, which is the DREB transcription factor binding site. This *cis*-regulatory element was found several times with varying flanking nucleotide sequences at *p*-values ranging from 1.2 *× *10^-5 ^to 4.3 *× *10^-5^.

#### Specific stress responses

**Figure 8:**
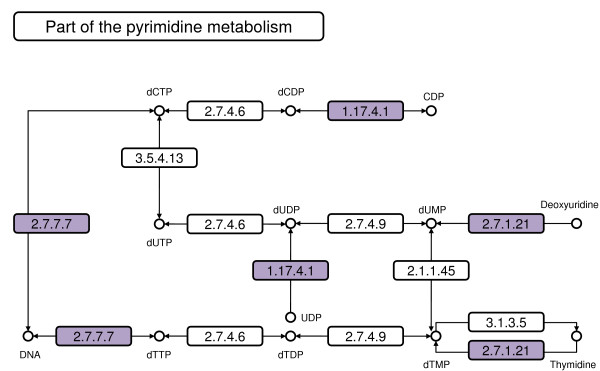
**Genotoxic stress genes involved in the pyrimidine metabolism**. The genes involved in the genotoxic stress module S23_*IR *_were mapped to the KEGG [49] pyrimidine metabolism of *Arabidopsis thaliana*. The enzymes that match the respective genes in the module S23_*IR *_are depicted in color. All these enzymes are involved in reactions catabolizing deoxynucleotides, which are precursors for the DNA synthesis.

Very pronounced responses can be found under exposure to oxidative stress in the shoot or salt stress in the root (Figure 6). One of the most striking patterns in this respect is the *single response *module S23_*IR *_which reacts to oxidative stress in the shoot tissue. A functional analysis of this cluster reveals an enrichment of the pyrimidine (*p*-value: 8.6 *× *10^-5^) and purine (*p*-value: 8.9 *× *10^-3^) metabolism. All mapped enzymes catalyze different deoxynucleotides, hence precursors necessary for DNA synthesis (see Figure 8). This finding is in accordance with the fact that module S23_*IR *_is a single response module, and thus only genotoxic stress has a significant effect on the DNA synthesis. Another *single response *module (R11_*IR*_) specifically responding under salt stress in the root is depicted in Figure 6. This is an especially interesting module, as the antisense of the W-box motif (TTGACTT) has been detected several times in the promoters. This is noteworthy, as the WRKY-transcription factors that bind to this element are already known to play a role in various abiotic and biotic stress responses. Two representatives of this class are contained within R11_*IR*_. The W-box motif AGTCAA has been found 96 times more frequently in the promoters of this dataset (*p*-value: 4.2 *× *10^-09^) than one would expect at random.

Overall, the *Arabidopsis thaliana *dataset contains *coherent*, *independent response *and *single response *modules (see Table 1). The *independent response *modules are the most numerous. The *coherent *modules occur less frequently. In the shoot tissue, these modules often contain cyclic genes which do not respond to the applied stresses. The *single response *modules occur several times, however not all the stresses could be related to such a module.

## Conclusion

Cells co-regulate the expression of their genes to respond appropriately to the sensed stimulus. They orchestrate these genes into general stress responses (*coherent *modules), some with different profiles (*independent response *modules), and into specific stress responses (*single response *modules). This modular response organization can only be observed in the light of multiple time-series microarray datasets. EDISA is capable of capturing such complex response patterns with manifold trajectories.

We evaluated the capability of EDISA to extract these response patterns from different datasets. Using a synthetic dataset, we showed that the algorithm is robust against noise and, despite its randomized nature, the results are rather stable. The EDISA could be applied to different biological datasets with the same parameter setting for *τ*_*G*_, *τ*_*C*_, and the number of iterations. The EDISA is capable of auto-adjusting the sensitive parameters during the iteration procedure. The predilection of the ISA approach for strong signals could be compensated for through the pre- and postprocessing procedures. This leads to a comprehensive set of distinct modules, covering a large variety of stress responses.

The subsequent analysis of these modules revealed interesting aspects of stress responses. This allowed the generation of hypotheses regarding the underlying regulatory system. For instance, observing general stress responses hints at a common regulatory control independent of the specific stress stimulus, while a regulatory mechanism responding to specific stress stimuli can be supported by *single response *modules. Such *single response *modules could also be captured by standard clustering methods. However, one can only distinguish between general and specific responses by finding these modules within 3D datasets. In the *Arabidopsis thaliana *dataset we have a strong indication of a common transcriptional control for osmotic and salt stress, whereas the *single response *genotoxic module hints at a specific response mechanism. In contrast, there has been only one stimulus applied to the *Homo sapiens *multiple sclerosis dataset. Therefore, differences in the response patterns of individual patients can be investigated, rather than differences in stimuli. The *Homo sapiens *multiple sclerosis modules exhibit a clear separation of patients into distinct groups, which respond differently to the same stimulus. These differences can be informative regarding disease states, disease progression and the respective regulatory mechanisms. The separation of the mathematical definition of a module and the mining algorithm allows for a flexible adaptation of both. The provided definition of the *independent response *modules captures several interesting responses, while remaining flexible with respect to the biological patterns and the structure of the dataset. Biological response patterns are allowed to be time-shifted or have completely different profiles. The dataset is not required to have the same number of measurements for every condition, or equal time-steps. Even the normalization protocol is not required to be equal. Hence, *independent response *modules can be used to analyze datasets from different experiments which cannot be compared directly. It is, however, important to stress that the biological validity of exploring this flexibility should be further addressed. Overall, the EDISA allows for a flexible, integrative analysis resulting in informative and dense modules, which can be subject to further downstream functional analysis.

## Methods

### Mathematical definitions

The gene expression matrix *E*^*GCT *^is composed of genes *G *= {*g*_1_, ⋯, *g*_|*G*|_}, conditions *C *= {*c*_1_, ⋯, *c*_|*C*|_} and time-points T={t1,⋯,t|Tc|}
 MathType@MTEF@5@5@+=feaafiart1ev1aaatCvAUfKttLearuWrP9MDH5MBPbIqV92AaeXatLxBI9gBaebbnrfifHhDYfgasaacH8akY=wiFfYdH8Gipec8Eeeu0xXdbba9frFj0=OqFfea0dXdd9vqai=hGuQ8kuc9pgc9s8qqaq=dirpe0xb9q8qiLsFr0=vr0=vr0dc8meaabaqaciaacaGaaeqabaqabeGadaaakeaacqWGubavcqGH9aqpdaGadeqaaiabdsha0naaBaaaleaacqaIXaqmaeqaaOGaeiilaWIaeS47IWKaeiilaWIaemiDaq3aaSbaaSqaamaaemaabaGaemivaq1aaSbaaWqaaiabdogaJbqabaaaliaawEa7caGLiWoaaeqaaaGccaGL7bGaayzFaaaaaa@3EDB@, were |*G*| denotes the number of genes, |*C*| the number of conditions and |*T*_*c*_| the number of time-points measured under the condition *c*. If |*T*_*c*_| varies throughout the dataset, only *independent response *modules can be mined, whereas, *coherent *and *independent response *modules can be mined in datasets where all conditions contain *d *samples. *E*^*gct *^refers to the expression value of gene *g *under condition *c *at time-point *t*. The vector **e**^*gcT*^specifies the profile of gene *g *under condition *c *over all time-points |*T*_*c*_| (equation 1). Using this notation, a row (**e**^*gCT*^), containing all conditions and time-points of a single gene, is defined in equation 2 and a column (**e**^*GcT*^), containing all genes and time-points under a single condition, in equation 3.

egcT=(Egct1,Egct2,...,Egct|Tc|)
 MathType@MTEF@5@5@+=feaafiart1ev1aaatCvAUfKttLearuWrP9MDH5MBPbIqV92AaeXatLxBI9gBaebbnrfifHhDYfgasaacH8akY=wiFfYdH8Gipec8Eeeu0xXdbba9frFj0=OqFfea0dXdd9vqai=hGuQ8kuc9pgc9s8qqaq=dirpe0xb9q8qiLsFr0=vr0=vr0dc8meaabaqaciaacaGaaeqabaqabeGadaaakeaaieqacqWFLbqzdaahaaWcbeqaaiabdEgaNjabdogaJjabdsfaubaakiabg2da9iabcIcaOiabdweafnaaCaaaleqabaGaem4zaCMaem4yamMaemiDaq3aaSbaaWqaaiabigdaXaqabaaaaOGaeiilaWIaemyrau0aaWbaaSqabeaacqWGNbWzcqWGJbWycqWG0baDdaWgaaadbaGaeGOmaidabeaaaaGccqGGSaalcqGGUaGlcqGGUaGlcqGGUaGlcqGGSaalcqWGfbqrdaahaaWcbeqaaiabdEgaNjabdogaJjabdsha0naaBaaameaadaabdaqaaiabdsfaunaaBaaabaGaem4yamgabeaaaiaawEa7caGLiWoaaeqaaaaakiabcMcaPaaa@5266@

egCT=(egc1T,egc2T,...,egc|C|T)
 MathType@MTEF@5@5@+=feaafiart1ev1aaatCvAUfKttLearuWrP9MDH5MBPbIqV92AaeXatLxBI9gBaebbnrfifHhDYfgasaacH8akY=wiFfYdH8Gipec8Eeeu0xXdbba9frFj0=OqFfea0dXdd9vqai=hGuQ8kuc9pgc9s8qqaq=dirpe0xb9q8qiLsFr0=vr0=vr0dc8meaabaqaciaacaGaaeqabaqabeGadaaakeaaieqacqWFLbqzdaahaaWcbeqaaiabdEgaNjabdoeadjabdsfaubaakiabg2da9iabcIcaOiab=vgaLnaaCaaaleqabaGaem4zaCMaem4yam2aaSbaaWqaaiabigdaXaqabaWccqWGubavaaGccqGGSaalcqWFLbqzdaahaaWcbeqaaiabdEgaNjabdogaJnaaBaaameaacqaIYaGmaeqaaSGaemivaqfaaOGaeiilaWIaeiOla4IaeiOla4IaeiOla4IaeiilaWIae8xzau2aaWbaaSqabeaacqWGNbWzcqWGJbWydaWgaaadbaWaaqWaaeaacqWGdbWqaiaawEa7caGLiWoaaeqaaSGaemivaqfaaOGaeiykaKcaaa@50A9@

eGcT=(eg1cT,eg2cT,...,eg|G|cT)′
 MathType@MTEF@5@5@+=feaafiart1ev1aaatCvAUfKttLearuWrP9MDH5MBPbIqV92AaeXatLxBI9gBaebbnrfifHhDYfgasaacH8akY=wiFfYdH8Gipec8Eeeu0xXdbba9frFj0=OqFfea0dXdd9vqai=hGuQ8kuc9pgc9s8qqaq=dirpe0xb9q8qiLsFr0=vr0=vr0dc8meaabaqaciaacaGaaeqabaqabeGadaaakeaaieqacqWFLbqzdaahaaWcbeqaaiabdEeahjabdogaJjabdsfaubaakiabg2da9iabcIcaOiab=vgaLnaaCaaaleqabaGaem4zaC2aaSbaaWqaaiabigdaXaqabaWccqWGJbWycqWGubavaaGccqGGSaalcqWFLbqzdaahaaWcbeqaaiabdEgaNnaaBaaameaacqaIYaGmaeqaaSGaem4yamMaemivaqfaaOGaeiilaWIaeiOla4IaeiOla4IaeiOla4IaeiilaWIae8xzau2aaWbaaSqabeaacqWGNbWzdaWgaaadbaWaaqWaaeaacqWGhbWraiaawEa7caGLiWoaaeqaaSGaem4yamMaemivaqfaaOGafiykaKIbauaaaaa@50BD@

Further, we define the average trajectory 〈eGmCmT〉
 MathType@MTEF@5@5@+=feaafiart1ev1aaatCvAUfKttLearuWrP9MDH5MBPbIqV92AaeXatLxBI9gBaebbnrfifHhDYfgasaacH8akY=wiFfYdH8Gipec8Eeeu0xXdbba9frFj0=OqFfea0dXdd9vqai=hGuQ8kuc9pgc9s8qqaq=dirpe0xb9q8qiLsFr0=vr0=vr0dc8meaabaqaciaacaGaaeqabaqabeGadaaakeaadaaadeqaaGqabiab=vgaLnaaCaaaleqabaGaem4raC0aaSbaaWqaaiabd2gaTbqabaWccqWGdbWqdaWgaaadbaGaemyBa0gabeaaliabdsfaubaaaOGaayzkJiaawQYiaaaa@369A@ over all conditions *C*_*m *_and all genes *G*_*m*_, as well as the average trajectory over all genes *G*_*m *_within one condition 〈eGmcT〉
 MathType@MTEF@5@5@+=feaafiart1ev1aaatCvAUfKttLearuWrP9MDH5MBPbIqV92AaeXatLxBI9gBaebbnrfifHhDYfgasaacH8akY=wiFfYdH8Gipec8Eeeu0xXdbba9frFj0=OqFfea0dXdd9vqai=hGuQ8kuc9pgc9s8qqaq=dirpe0xb9q8qiLsFr0=vr0=vr0dc8meaabaqaciaacaGaaeqabaqabeGadaaakeaadaaadeqaaGqabiab=vgaLnaaCaaaleqabaGaem4raC0aaSbaaWqaaiabd2gaTbqabaWccqWGJbWycqWGubavaaaakiaawMYicaGLQmcaaaa@353F@. Each trajectory contributing to the average is assigned a weight. This weight is specified for each gene and condition by the vectors *W*^*G *^and *W*^*C*^. The denominator normalizes the average profile in accordance to these weight vectors:

〈eGmCmT〉=∑g∈GmwgG⋅∑c∈CmwcC⋅egcT‖wCmC‖1⋅‖wGmG‖1
 MathType@MTEF@5@5@+=feaafiart1ev1aaatCvAUfKttLearuWrP9MDH5MBPbIqV92AaeXatLxBI9gBaebbnrfifHhDYfgasaacH8akY=wiFfYdH8Gipec8Eeeu0xXdbba9frFj0=OqFfea0dXdd9vqai=hGuQ8kuc9pgc9s8qqaq=dirpe0xb9q8qiLsFr0=vr0=vr0dc8meaabaqaciaacaGaaeqabaqabeGadaaakeaadaaadeqaaGqabiab=vgaLnaaCaaaleqabaGaem4raC0aaSbaaWqaaiabd2gaTbqabaWccqWGdbWqdaWgaaadbaGaemyBa0gabeaaliabdsfaubaaaOGaayzkJiaawQYiaiabg2da9maalaaabaWaaabeaeaacqWG3bWDdaqhaaWcbaGaem4zaCgabaGaem4raCeaaOGaeyyXICnaleaacqWGNbWzcqGHiiIZcqWGhbWrdaWgaaadbaGaemyBa0gabeaaaSqab0GaeyyeIuoakmaaqababaGaem4DaC3aa0baaSqaaiabdogaJbqaaiabdoeadbaakiabgwSixlab=vgaLnaaCaaaleqabaGaem4zaCMaem4yamMaemivaqfaaaqaaiabdogaJjabgIGiolabdoeadnaaBaaameaacqWGTbqBaeqaaaWcbeqdcqGHris5aaGcbaWaauWaaeaacqWG3bWDdaqhaaWcbaGaem4qam0aaSbaaWqaaiabd2gaTbqabaaaleaacqWGdbWqaaaakiaawMa7caGLkWoadaWgaaWcbaGaeGymaedabeaakiabgwSixpaafmaabaGaem4DaC3aa0baaSqaaiabdEeahnaaBaaameaacqWGTbqBaeqaaaWcbaGaem4raCeaaaGccaGLjWUaayPcSdWaaSbaaSqaaiabigdaXaqabaaaaaaa@6E50@

〈eGmcT〉=∑g∈GmwgG⋅egcT‖wGmG‖1
 MathType@MTEF@5@5@+=feaafiart1ev1aaatCvAUfKttLearuWrP9MDH5MBPbIqV92AaeXatLxBI9gBaebbnrfifHhDYfgasaacH8akY=wiFfYdH8Gipec8Eeeu0xXdbba9frFj0=OqFfea0dXdd9vqai=hGuQ8kuc9pgc9s8qqaq=dirpe0xb9q8qiLsFr0=vr0=vr0dc8meaabaqaciaacaGaaeqabaqabeGadaaakeaadaaadeqaaGqabiab=vgaLnaaCaaaleqabaGaem4raC0aaSbaaWqaaiabd2gaTbqabaWccqWGJbWycqWGubavaaaakiaawMYicaGLQmcacqGH9aqpdaWcaaqaamaaqababaGaem4DaC3aa0baaSqaaiabdEgaNbqaaiabdEeahbaaaeaacqWGNbWzcqGHiiIZcqWGhbWrdaWgaaadbaGaemyBa0gabeaaaSqab0GaeyyeIuoakiabgwSixlab=vgaLnaaCaaaleqabaGaem4zaCMaem4yamMaemivaqfaaaGcbaWaauWaaeaacqWG3bWDdaqhaaWcbaGaem4raC0aaSbaaWqaaiabd2gaTbqabaaaleaacqWGhbWraaaakiaawMa7caGLkWoadaWgaaWcbaGaeGymaedabeaaaaaaaa@5337@

To quantify the similarity of two genes we apply the Pearson distance *ρ *to their profile. This distance can be related to the Pearson correlation coefficient *r *by the simple formula *ρ *= 1 - *r*. Now, we can provide a mathematical definition for the *coherent *and *independent response *modules, where *M *refers to a module containing the genes *G*_*M *_and the conditions *C*_*M*_.

#### Coherent modules

For the *coherent *modules the Pearson distance of each condition *c*, containing the genes *G*_*m*_, to the average trajectory (equation 4) must not exceed the threshold *τ*_*C*_. Accordingly, the Pearson distance of every gene *g*, under the conditions *C*_*m*_, to the average trajectory (equation 4) must not exceed the threshold *τ*_*G*_. Coherent modules are defined as follows:

Mco(τC,τG):={(Gm,Cm)|∀c∈Cm:1|Gm|⋅∑g∈Gmρ(egcT,〈eGmCmT〉)<τC∀g∈Gm:1|Cm|⋅∑c∈Cmρ(egcT,〈eGmCmT〉)<τG}
 MathType@MTEF@5@5@+=feaafiart1ev1aaatCvAUfKttLearuWrP9MDH5MBPbIqV92AaeXatLxBI9gBaebbnrfifHhDYfgasaacH8akY=wiFfYdH8Gipec8Eeeu0xXdbba9frFj0=OqFfea0dXdd9vqai=hGuQ8kuc9pgc9s8qqaq=dirpe0xb9q8qiLsFr0=vr0=vr0dc8meaabaqaciaacaGaaeqabaqabeGadaaakeaacqWGnbqtdaahaaWcbeqaaiabdogaJjabd+gaVbaakiabcIcaOGGaciab=r8a0naaBaaaleaacqWGdbWqaeqaaOGaeiilaWIae8hXdq3aaSbaaSqaaiabdEeahbqabaGccqGGPaqkcqGG6aGocqGH9aqpdaGadeqaaiabcIcaOiabdEeahnaaBaaaleaacqWGTbqBaeqaaOGaeiilaWIaem4qam0aaSbaaSqaaiabd2gaTbqabaGccqGGPaqkdaabbaqaauaabeqaciaaaeaacqGHaiIicqWGJbWycqGHiiIZcqWGdbWqdaWgaaWcbaGaemyBa0gabeaakiabcQda6maalaaabaGaeGymaedabaWaaqWaaeaacqWGhbWrdaWgaaWcbaGaemyBa0gabeaaaOGaay5bSlaawIa7aaaacqGHflY1daaeqaqaaiab=f8aYnaabmaabaacbeGae4xzau2aaWbaaSqabeaacqWGNbWzcqWGJbWycqWGubavaaGccqGGSaaldaaadeqaaiab+vgaLnaaCaaaleqabaGaem4raC0aaSbaaWqaaiabd2gaTbqabaWccqWGdbWqdaWgaaadbaGaemyBa0gabeaaliabdsfaubaaaOGaayzkJiaawQYiaaGaayjkaiaawMcaaaWcbaGaem4zaCMaeyicI4Saem4raC0aaSbaaWqaaiabd2gaTbqabaaaleqaniabggHiLdaakeaacqGH8aapcqWFepaDdaWgaaWcbaGaem4qameabeaaaOqaaiabgcGiIiabdEgaNjabgIGiolabdEeahnaaBaaaleaacqWGTbqBaeqaaOGaeiOoaOZaaSaaaeaacqaIXaqmaeaadaabdaqaaiabdoeadnaaBaaaleaacqWGTbqBaeqaaaGccaGLhWUaayjcSdaaaiabgwSixpaaqababaGae8xWdihaleaacqWGJbWycqGHiiIZcqWGdbWqdaWgaaadbaGaemyBa0gabeaaaSqab0GaeyyeIuoakmaabmaabaGae4xzau2aaWbaaSqabeaacqWGNbWzcqWGJbWycqWGubavaaGccqGGSaaldaaadeqaaiab+vgaLnaaCaaaleqabaGaem4raC0aaSbaaWqaaiabd2gaTbqabaWccqWGdbWqdaWgaaadbaGaemyBa0gabeaaliabdsfaubaaaOGaayzkJiaawQYiaaGaayjkaiaawMcaaaqaaiabgYda8iab=r8a0naaBaaaleaacqWGhbWraeqaaaaaaOGaay5bSdaacaGL7bGaayzFaaaaaa@A5CA@

#### Independent response modules

In case we wish to mine *independent response *modules instead of *coherent *modules, comparisons are restricted to be within conditions. This restriction is imposed because comparisons between conditions are only desirable if one wants to align the profile of different conditions, as it is done for the *coherent *modules. Thus, for each condition *c *∈ *C*_*m *_we average over the Pearson distance of each trajectory contained in *G*_*m *_to the average trajectory of all genes contained in *G*_*m *_(equation 5). This average must not exceed the threshold *τ*_*C*_. Accordingly, for each gene *g *∈ *G*_*m *_we average over the distance of each trajectory contained in *C*_*m *_to the average trajectory of all genes contained in *G*_*m*_. This average must not exceed the threshold *τ*_*G*_. *Independent response *modules are defined as follows:

Mir(τC,τG):={(Gm,Cm)|∀c∈Cm:1|Gm|⋅∑g∈Gmρ(egcT,〈eGmcT〉)<τC∀g∈Gm:1|Cm|⋅∑c∈Cmρ(egcT,〈eGmcT〉)<τG}
 MathType@MTEF@5@5@+=feaafiart1ev1aaatCvAUfKttLearuWrP9MDH5MBPbIqV92AaeXatLxBI9gBaebbnrfifHhDYfgasaacH8akY=wiFfYdH8Gipec8Eeeu0xXdbba9frFj0=OqFfea0dXdd9vqai=hGuQ8kuc9pgc9s8qqaq=dirpe0xb9q8qiLsFr0=vr0=vr0dc8meaabaqaciaacaGaaeqabaqabeGadaaakeaacqWGnbqtdaahaaWcbeqaaiabdMgaPjabdkhaYbaakiabcIcaOGGaciab=r8a0naaBaaaleaacqWGdbWqaeqaaOGaeiilaWIae8hXdq3aaSbaaSqaaiabdEeahbqabaGccqGGPaqkcqGG6aGocqGH9aqpdaGadeqaaiabcIcaOiabdEeahnaaBaaaleaacqWGTbqBaeqaaOGaeiilaWIaem4qam0aaSbaaSqaaiabd2gaTbqabaGccqGGPaqkdaabbaqaauaabeqaciaaaeaacqGHaiIicqWGJbWycqGHiiIZcqWGdbWqdaWgaaWcbaGaemyBa0gabeaakiabcQda6maalaaabaGaeGymaedabaWaaqWaaeaacqWGhbWrdaWgaaWcbaGaemyBa0gabeaaaOGaay5bSlaawIa7aaaacqGHflY1daaeqaqaaiab=f8aYnaabmaabaacbeGae4xzau2aaWbaaSqabeaacqWGNbWzcqWGJbWycqWGubavaaGccqGGSaaldaaadeqaaiab+vgaLnaaCaaaleqabaGaem4raC0aaSbaaWqaaiabd2gaTbqabaWccqWGJbWycqWGubavaaaakiaawMYicaGLQmcaaiaawIcacaGLPaaaaSqaaiabdEgaNjabgIGiolabdEeahnaaBaaameaacqWGTbqBaeqaaaWcbeqdcqGHris5aaGcbaGaeyipaWJae8hXdq3aaSbaaSqaaiabdoeadbqabaaakeaacqGHaiIicqWGNbWzcqGHiiIZcqWGhbWrdaWgaaWcbaGaemyBa0gabeaakiabcQda6maalaaabaGaeGymaedabaWaaqWaaeaacqWGdbWqdaWgaaWcbaGaemyBa0gabeaaaOGaay5bSlaawIa7aaaacqGHflY1daaeqaqaaiab=f8aYbWcbaGaem4yamMaeyicI4Saem4qam0aaSbaaWqaaiabd2gaTbqabaaaleqaniabggHiLdGcdaqadaqaaiab+vgaLnaaCaaaleqabaGaem4zaCMaem4yamMaemivaqfaaOGaeiilaWYaaaWabeaacqGFLbqzdaahaaWcbeqaaiabdEeahnaaBaaameaacqWGTbqBaeqaaSGaem4yamMaemivaqfaaaGccaGLPmIaayPkJaaacaGLOaGaayzkaaaabaGaeyipaWJae8hXdq3aaSbaaSqaaiabdEeahbqabaaaaaGccaGLhWoaaiaawUhacaGL9baaaaa@A326@

#### Single response modules

As a special case of *coherent *or *independent response *modules, the *single response *module type can be defined by setting the number of conditions to one.

Note, that all definitions are symmetric with respect to the genes and conditions. For the *independent response *modules no assumption is made regarding the comparability of the expression values among the conditions. This enables the comparison of experiments with different dimensions, time intervals, and normalization protocols.

### Preprocessing

Prior to the preprocessing procedure, genes are filtered from the dataset if they do not have a two-fold difference under at least one condition. This step aims at removing noise signals and unaffected genes. For the *Arabidopsis thaliana *dataset, a control measurement was available as reference point. For the *Homo sapiens *multiple sclerosis dataset no control measurements were available. Here we used the first measurement as a reference for all time-points.

EDISA is designed to refine initial modules sampled from prefiltered datasets. Such modules could be randomly drawn from the dataset. However, this leads to a predilection for strong signals, which is a recognized problem of the ISA approach. Therefore, before applying the module refinement, samples are drawn from the dataset with the aim of creating initial modules which follow a certain trajectory representing the signal of a module, while the set of all samples covers a broad range of signals. The approach proceeds by randomly sampling seed genes and, according to the Pearson distance, selecting its *s *- 1 nearest neighbors within one of the conditions, where *s *is the desired sample size. To cover a broad range of signals we are not interested in drawing the same genes too frequently. Therefore, we draw genes without replacement to obtain module samples of size *s *= 30.

### Module scores

The mathematical definitions of the modules specify the set of all modules of a desired type. However, to mine for modules contained in the set, we need to define a scoring function, in accordance with the module definition. This scoring function is employed throughout the iterative procedure of the EDISA algorithm. Analogous to the *coherent *module definition, the scoring function for *coherent *modules is defined:

Sco(GmCmT)=1|Gm||Cm|∑g∈Gm,c∈Cmρ(egcT,〈eGmCmT〉)
 MathType@MTEF@5@5@+=feaafiart1ev1aaatCvAUfKttLearuWrP9MDH5MBPbIqV92AaeXatLxBI9gBaebbnrfifHhDYfgasaacH8akY=wiFfYdH8Gipec8Eeeu0xXdbba9frFj0=OqFfea0dXdd9vqai=hGuQ8kuc9pgc9s8qqaq=dirpe0xb9q8qiLsFr0=vr0=vr0dc8meaabaqaciaacaGaaeqabaqabeGadaaakeaacqWGtbWudaWgaaWcbaGaem4yamMaem4Ba8gabeaakiabcIcaOiabdEeahnaaBaaaleaacqWGTbqBaeqaaOGaem4qam0aaSbaaSqaaiabd2gaTbqabaGccqWGubavcqGGPaqkcqGH9aqpdaWcaaqaaiabigdaXaqaamaaemaabaGaem4raC0aaSbaaSqaaiabd2gaTbqabaaakiaawEa7caGLiWoadaabdaqaaiabdoeadnaaBaaaleaacqWGTbqBaeqaaaGccaGLhWUaayjcSdaaamaaqafabaacciGae8xWdi3aaeWaaeaaieqacqGFLbqzdaahaaWcbeqaaiabdEgaNjabdogaJjabdsfaubaakiabcYcaSmaaamqabaGae4xzau2aaWbaaSqabeaacqWGhbWrdaWgaaadbaGaemyBa0gabeaaliabdoeadnaaBaaameaacqWGTbqBaeqaaSGaemivaqfaaaGccaGLPmIaayPkJaaacaGLOaGaayzkaaaaleaacqWGNbWzcqGHiiIZcqWGhbWrdaWgaaadbaGaemyBa0gabeaaliabcYcaSiabdogaJjabgIGiolabdoeadnaaBaaameaacqWGTbqBaeqaaaWcbeqdcqGHris5aaaa@682A@

Analogous to the *independent response *module definition, the scoring function for *independent response *modules is defined as follows:

Sir(GmCmT)=1|Gm||Cm|∑g∈Gm,c∈Cmρ(egcT,〈eGmcT〉)
 MathType@MTEF@5@5@+=feaafiart1ev1aaatCvAUfKttLearuWrP9MDH5MBPbIqV92AaeXatLxBI9gBaebbnrfifHhDYfgasaacH8akY=wiFfYdH8Gipec8Eeeu0xXdbba9frFj0=OqFfea0dXdd9vqai=hGuQ8kuc9pgc9s8qqaq=dirpe0xb9q8qiLsFr0=vr0=vr0dc8meaabaqaciaacaGaaeqabaqabeGadaaakeaacqWGtbWudaWgaaWcbaGaemyAaKMaemOCaihabeaakiabcIcaOiabdEeahnaaBaaaleaacqWGTbqBaeqaaOGaem4qam0aaSbaaSqaaiabd2gaTbqabaGccqWGubavcqGGPaqkcqGH9aqpdaWcaaqaaiabigdaXaqaamaaemaabaGaem4raC0aaSbaaSqaaiabd2gaTbqabaaakiaawEa7caGLiWoadaabdaqaaiabdoeadnaaBaaaleaacqWGTbqBaeqaaaGccaGLhWUaayjcSdaaamaaqafabaacciGae8xWdi3aaeWaaeaaieqacqGFLbqzdaahaaWcbeqaaiabdEgaNjabdogaJjabdsfaubaakiabcYcaSmaaamqabaGae4xzau2aaWbaaSqabeaacqWGhbWrdaWgaaadbaGaemyBa0gabeaaliabdogaJjabdsfaubaaaOGaayzkJiaawQYiaaGaayjkaiaawMcaaaWcbaGaem4zaCMaeyicI4Saem4raC0aaSbaaWqaaiabd2gaTbqabaWccqGGSaalcqWGJbWycqGHiiIZcqWGdbWqdaWgaaadbaGaemyBa0gabeaaaSqab0GaeyyeIuoaaaa@66E1@

### Iteration

At each iteration step *i *the iteration scheme applies a filter to remove those genes and conditions from *G*^*i *^and *C*^*i*^, which do not meet the module criteria. This results in new gene and condition sets *G*^*i*+1 ^and *C*^*i*+1^, for the next iteration step *i *+ 1. In order to explicitly mine for either *coherent *or *independent response *modules, the score of each module is computed with the respective scoring function. This procedure implies that genes and conditions are treated equally.

Assume, we are searching for *coherent *modules. Then, given the current *G*^*i *^and *C*^*i*^, the score for each gene and condition in the module is computed using the scoring function *S*_*co*_:

*C*^*i*+1 ^= {∀*c *∈ *C*^*i*^|*S*_*co *_(*G*^*i*^*cT*) <*τ*_*C*_}

Given *G*^*i *^and *C*^*i*+1 ^the next iteration step is:

*G*^*i*+1 ^= {∀*g *∈ *G*^*i*^|*S*_*co *_(*gC*^*i*+1^*T*) <*τ*_*G*_}

To search for *independent response *modules, the scoring function *S*_*ir *_is applied.

*C*^*i*+1 ^= {∀*c *∈ *C*^*i*^|*S*_*ir *_(*G*^*i*^*cT *<*τ*_*C*_}

Given *G*^*i *^and *C*^*i*+1 ^the iteration step is:

*G*^*i*+1 ^= {∀*g *∈ *G*^*i*^|*S*_*ir *_(*gC*^*i*+1^*T*) <*τ*_*G*_}

These iteration formulas are repeated until *G*^*i *^= *G*^*i*+1 ^and *C*^*i *^= *C*^*i*+1 ^holds.

#### Average trajectory calculation

The initial sample drawn by the preprocessing step has a fixed size *s*. Often, the size of this sample is larger than the number of genes contained in a module. Thus a small signal is embedded into a relatively large amount of background noise, which is likely to dominate the average. To account for this effect, a weighted average trajectory is used (equation 4), which takes advantage of the preprocessing procedure. Thereby, *s *genes are selected iteratively, where at every step the gene with the smallest Pearson distance to the seed gene *g *is added. To assign a weight to each gene, we generate a weighting vector *W*^*G *^by drawing *s *samples from the exponential distribution with *λ *= 1. These weights are sorted and the highest weight is associated with the seed gene *g*. Then they descend according to their Pearson distance to *g *from 1 to *s*.

#### Automatically refining the parameters *τ*_*C *_and *τ*_*G*_

The mathematical definition of the modules defines a set of modules by applying global thresholds *τ*_*G *_and *τ*_*C*_. However, initial modules are often fuzzy and contain random genes, disrupting the average of the final module. Thus, initially a less restrictive threshold is needed, which, as the iteration proceeds, can be decreased to narrow the module down to its dense core. This refinement is only employed during the iteration procedure and does not affect *τ*_*G *_and *τ*_*C *_in the postprocessing.

The adaptation of *τ*_*C *_and *τ*_*G *_is accomplished by applying a *k*-means clustering with *k *= 2 at each iteration step. Thus, *k*-means separates the genes or conditions into two sets, one which should remain in the module and another which should be discarded. For both sets the module definitions are applied to calculate the minimal acceptable values of *τ*_*G *_and *τ*_*C*_. The new thresholds are then set to the minimal *τ*_*G *_and *τ*_*C*_, respectively. Given these thresholds, the iteration formula refines the modules in accordance with the clusters determined by *k*-means. The thresholds are left unaffected if *k*-means is unable to partition the modules.

This adaptation procedure increased the performance of EDISA significantly on the synthetic dataset.

### Postprocessing

The EDISA approach draws a large number of random samples. It is inevitable that this approach can yield the same module a number of times. Furthermore, a maximal module may be found along with numerous copies of its submodules. Consequently, for a proper evaluation of the results, the sampled modules are merged.

First the merging procedure filters out all modules with a value above a specified threshold *τ *(equation 8 or 9). Then, a *k*-means clustering, with 10 restarts, is performed on the remaining modules. The clustering operates on the pairwise Pearson distances of the centroids, so that similar centroids are clustered. The parameter *k *is set to the number of principal components which explain for 95% of the variation in the centroid distance matrix. All modules that cluster together are tested for inclusion and all modules are discarded which are subsets of other modules. This inclusion test could also be performed without clustering the modules, however, the clustering procedure provides a significant runtime improvement. The merged modules are refined by two extension steps. The first extension step considers all genes in the dataset and adds them to the module if their correlation to the average module trajectory is below the threshold *τ*_*G*_. Accordingly, the second extension step considers for every condition whether it should be added to the module, by matching its average correlation against *τ*_*C*_. Both extension steps are carried out in accordance with equations 6 and 7. After the extension step a final filter is applied, removing all modules which have an overlap (according to equation 14) of more than 75% with another module.

### Organization of the modules

A requirement for module definitions is that the modules are allowed to overlap. To visualize the number of genes shared by different modules, we represent their relationship by a graph, in which the edges indicate the degree of overlap between two modules. The edge weight is calculated by equation 14. A weight of 0 indicates no overlap and 1 indicates module identity. Edges with a value below 0.15 are not drawn.

DM(M1,M2)=|G1∩G2||G1∪G2|
 MathType@MTEF@5@5@+=feaafiart1ev1aaatCvAUfKttLearuWrP9MDH5MBPbIqV92AaeXatLxBI9gBaebbnrfifHhDYfgasaacH8akY=wiFfYdH8Gipec8Eeeu0xXdbba9frFj0=OqFfea0dXdd9vqai=hGuQ8kuc9pgc9s8qqaq=dirpe0xb9q8qiLsFr0=vr0=vr0dc8meaabaqaciaacaGaaeqabaqabeGadaaakeaacqWGebardaWgaaWcbaGaemyta0eabeaakiabcIcaOiabd2eannaaBaaaleaacqaIXaqmaeqaaOGaeiilaWIaemyta00aaSbaaSqaaiabikdaYaqabaGccqGGPaqkcqGH9aqpdaWcaaqaamaaemaabaGaem4raC0aaSbaaSqaaiabigdaXaqabaGccqGHPiYXcqWGhbWrdaWgaaWcbaGaeGOmaidabeaaaOGaay5bSlaawIa7aaqaamaaemaabaGaem4raC0aaSbaaSqaaiabigdaXaqabaGccqGHQicYcqWGhbWrdaWgaaWcbaGaeGOmaidabeaaaOGaay5bSlaawIa7aaaaaaa@49CC@

### Evaluation on the synthetic dataset

To evaluate the EDISA approach, we applied it to a synthetic dataset with implanted modules. To obtain a score for recovered modules, each module *M *is compared against the most similar implanted module (equation 15). A perfect correspondence of the recovered and implanted modules results in a score of 1, whereas completely disjoint modules score with 0. The equation employed for this score is similar to the previously introduced postprocessing equation (equation 14). Two sets of modules *R*_1 _= (*M*_1_, ... *M*_|*R*1|_) and *R*_2 _= (*M*_1_, ... *M*_|*R*2|_) are compared based on the genes *G*_*n *_and conditions *C*_*n *_which are part of the respective modules.

S∗(R1,R2)=∑G1,C1∈R1max⁡G2,C2∈R2S(G1,G2)|R1|
 MathType@MTEF@5@5@+=feaafiart1ev1aaatCvAUfKttLearuWrP9MDH5MBPbIqV92AaeXatLxBI9gBaebbnrfifHhDYfgasaacH8akY=wiFfYdH8Gipec8Eeeu0xXdbba9frFj0=OqFfea0dXdd9vqai=hGuQ8kuc9pgc9s8qqaq=dirpe0xb9q8qiLsFr0=vr0=vr0dc8meaabaqaciaacaGaaeqabaqabeGadaaakeaacqWGtbWudaahaaWcbeqaaiabgEHiQaaakiabcIcaOiabdkfasnaaBaaaleaacqaIXaqmaeqaaOGaeiilaWIaemOuai1aaSbaaSqaaiabikdaYaqabaGccqGGPaqkcqGH9aqpdaWcaaqaamaaqababaGagiyBa0MaeiyyaeMaeiiEaG3aaSbaaSqaaiabdEeahnaaBaaameaacqaIYaGmaeqaaSGaeiilaWIaem4qam0aaSbaaWqaaiabikdaYaqabaWccqGHiiIZcqWGsbGudaWgaaadbaGaeGOmaidabeaaaSqabaaabaGaem4raC0aaSbaaWqaaiabigdaXaqabaWccqGGSaalcqWGdbWqdaWgaaadbaGaeGymaedabeaaliabgIGiolabdkfasnaaBaaameaacqaIXaqmaeqaaaWcbeqdcqGHris5aOGaem4uamLaeiikaGIaem4raC0aaSbaaSqaaiabigdaXaqabaGccqGGSaalcqWGhbWrdaWgaaWcbaGaeGOmaidabeaakiabcMcaPaqaamaaemaabaGaemOuai1aaSbaaSqaaiabigdaXaqabaaakiaawEa7caGLiWoaaaaaaa@5D9B@

### Analysis of cis-regulatory elements

The analysis on *cis*-regulatory elements has been carried out with the RSA-tools package described by van Helden *et al*. [45,46], which is based on the frequency of a motif over its respective background frequency. Motifs found significantly enriched in the 1100 bp upstream region of the translation start site (ATG) were subsequently compared to the PLACE database [47] to identify motifs of known regulatory function.

### Datasets

#### Synthetic

The synthetic dataset contains 1,000 genes measured over 10 conditions with 6 time-points each. The measurements were generated by drawing the first time-point from a normal distribution with a mean of 5 and a variance of 0.3. The remaining time-points were sampled from a normal distribution with a variance of 0.3 and a mean centered at the first time-point. Into this background model four modules were implanted. Each module contained 50 genes and three of the modules were overlapping. In the case of perfectly correlated modules, the noise level within the modules is *σ *= 0. To introduce artificial variance to the modules, noise was added to the modules by a normal distribution with different standard deviations *σ *= (0.1, 0.3, 0.5, 0.7, 0.9) [see additional file 1].

### Homo sapiens multiple sclerosis dataset

The dataset was generated in the course of a pharmacological study analyzing the response of multiple sclerosis patients to IFN-*β *treatment [25]. Peripheral blood of 14 multiple sclerosis patients was obtained and the measurements were conducted before as well as 1, 2, 4, 8, 24, 48, 120, and 168 h after the treatment. This dataset was obtained from the authors of the Guttman *et al*. publication [25].

### Arabidopsis thaliana abiotic stress dataset

The *Arabidopsis thaliana *dataset is provided by the AFGN (Arabidopsis Functional Genomics Network) and available at TAIR [22]. Within this project, 9 time-series experiments were conducted [26]. Among these, we extracted a group of abiotic stress stimuli for the tissues shoot and root, as well as the respective control measurements. The stress conditions and their reference numbers at TAIR are (cold: ME00325, osmotic: ME00327, salt: ME00328, drought: ME00338, genotoxic: ME00326, uv-b: ME00329, wound: ME00330, heat: ME00339). Each of these time-series contains 6 to 9 measurements with two biological replicates.

The signals were normalized with GCRMA [48], the biological replicates were averaged and finally the log_2 _was taken.

## Competing interests

The authors declare that they have no competing interests.

## Authors' contributions

JS wrote the manuscript. JS and MS developed the mathematical methods and algorithm. MS implemented the mathematical model in MATLAB™. DW made major contributions towards the biological interpretation of the results. AZ and KH were involved in study design and coordination. All authors read and approved the final manuscript.

## Supplementary Material

Additional file 2 Gene lists of all modules. Contains the gene lists for all modules extracted from the *Arabidopsis thaliana *and *Homo sapiens *dataset. Click here for file

Additional file 1 Synthetic dataset. The synthetic dataset used for the evaluation analysis. Click here for file
